# B12 Deficiency and Clinical Presentation in the Setting of Nitric Oxide Use

**DOI:** 10.1155/2021/5590948

**Published:** 2021-04-08

**Authors:** Donald Mario Robert Harker, Bridget Martinez, Burton J. Tabaac

**Affiliations:** ^1^St. George's Medical University, School of Medicine, Grenada, Grenada; ^2^Department of Pharmacology, University of Nevada, Reno School of Medicine, Reno, NV, USA; ^3^Department of Neurology, University of Nevada, Reno School of Medicine, Reno, NV, USA; ^4^Acute Care Neurology Division, Renown Regional Medical Center, Reno, NV, USA

## Abstract

B12 deficiency can arise symptomatically from an array of varying pathologies including frank deficiency from strict vegan diets. Other high-risk contributing pathological conditions include chronic alcoholism, autoimmune disease, and chronic gastrointestinal inflammatory disorders, and it is also seen in those with a history of gastric surgery. Additionally, the elderly are at an increased risk as are patients prescribed certain medications. Uncommonly suspected causes of B12 deficiency include the abuse of recreational nitrous oxide (NO) given its interference with cobalt oxidation. Here, we report two cases of hypovitaminosis B12 in association with NO abuse in an effort to highlight an increasingly dangerous trend with recreational use. Importantly, we aim to increase visibility of this malady given that improperly diagnosed neurologic deterioration following NO anesthesia has been shown to become irreversible and may even result in death.

## 1. Introduction

Cobalamin, also known as vitamin B12, is an essential and natural water-soluble vitamin necessary in a variety of homeostatic functions [1]. The range of signs and symptoms related to the deficient state can be quite heterogeneous due to the many metabolic processes it is involved in including the conversion of L-methylmalonyl coenzyme A into succinyl coenzyme A and the formation of methionine by methylation of homocysteine [2]. These reactions are vitally necessary as the first supplements the Krebs cycle and hemopoiesis and the latter allows recovery of THF necessary for the biogenesis of nucleic acids [3]. The commonly associated presentation is a megaloblastic anemia, with subacute combined degeneration of the spinal cord as the worst-case outcome, characterized by degeneration of the dorsal columns and the lateral columns secondary to demyelination [4]. Given that presentation can vary and is diverse from patient to patient, it is difficult to suspect vitamin B12 deficiency in patients presenting without anemia, in those presenting with initial psychiatric manifestations, or other atypical presentations [5]. However, why presenting symptoms vary so greatly is not understood, though it may be related to the specificity of the affected pathway. Deficiency can occur through a variety of means including poor intake, pancreatic disease, medication, problems of absorption, genetic disease, and chemical inactivation from compounds such as nitrous oxide [5–8].

Nitrous oxide (NO) is a commonly used inhaled anesthetic which provides an acute state of euphoria, depersonalization, derealization, anxiolytic effects, and anesthesia, regularly described by laypersons as “laughing gas” and “whippets” or “hippy crack” in the recreational community [9]. Administration of NO can be performed alone or as an adjunct in a variety of settings ranging from general anesthesia, procedural settings, or for pain control usually through a face mask or secured advanced airway. NO's mechanism of action involves noncompetitive NMDA inhibition in the central nervous system, secretion of endogenous opioids, and GABA-A stimulation [10]. Symptomatic disease related to NO use is most commonly observed in the setting of recreational use as abusers have a higher potential for prolonged use [11]. The lifetime prevalence of abuse ranges from 2% to 15.8% in the younger population, elevated in males who live in rural environments; though these numbers may be lower than the true rate, reported cases of nitrous oxide abuse continue to rise [11]. Given that the active form of vitamin B12 contains cobalt in its reduced form (Co+) and given that NO promotes the irreversible oxidation of Co+ to the Co++ and the Co forms, chemically, NO abuse renders vitamin B12 inactive [12].

Although vitamin B12 deficiency is usually suspected clinically, a definitive diagnosis can only be made through blood labs measuring serum levels of vitamin B12. Serum vitamin B12 levels below 148 pmol/l are highly sensitive, between 95–97%, for hypovitaminosis B12 in the context of a symptomatic patient. Associated labs include serum levels of methylmalonic acid (MMA) level and folate (1, 2). Elevated serum MMA levels point towards a backup in B12 metabolic pathway metabolites. The buildup of MMA is >95% sensitive for hypovitaminosis B12 representing a low substrate availability in methionine synthase's ability to regenerate methionine and tetrahydrofolate by reacting homocysteine with N5-methyltetrahydrofolate (1, 2, 3).

Screening the general population for hypovitaminosis B12 levels is not currently recommended; however, a laboratory workup should be considered on a case-by-case basis when a patient presents with symptoms and/or has a history of known high-risk factors. The approach to treatment should be based on the patient's individual presentation and underlying cause [13]. Asymptomatic patients with serum vitamin B12 levels between 150 to 399 pg/ml may be treated with 1 mg intramuscular administration of cyanocobalamin three times weekly for two weeks according to the British Society for Haematology or oral supplementation, based on patient preference, with follow-up testing only required if the patient develops further complications [13]. It should be noted that all symptomatic patients should be treated with intramuscular injections of cyanocobalamin if available as this allows for body stores of B12 to be restored expeditiously and reestablishes the functional metabolic pathways involving B12 [13]. In the setting of noted neurologic deficits, intramuscular injections should be administered quaque alternis die (QOD), or every 48 hours, for at least 3 weeks [13]. Administration of IM B12 should be continued past the three-week mark until there is no longer discernible recovery of the patient's neurologic condition (plateau in recovery).

Hypovitaminosis B12 is a pervasive condition globally, with a prevalence that rises with increasing age with approximately 3% of the population affected between 20 and 39 and increasing to 6% of the population affected in those over 60 years of age [14]. The human body retains an incredible capacity for hepatic vitamin B12 storage allowing for 5 to 10 years of insufficient intake prior to the appearance of symptomatic deficiency [15]. Populations that should raise the index of suspicion for hypovitaminosis B12 include those that adhere to a strict vegan diet, chronic alcoholics, those with a history of autoimmune disease or chronic gastrointestinal inflammatory disorders, postgastric surgery, family history, the elderly, patients on certain medications, and those with a history of recreational NO abuse. Here, we present two cases of symptomatic hypovitaminosis B12 in the setting of NO abuse.

## 2. Case 1

A 38-year-old-male with a past medical history significant for acid reflux, pancreatitis, and substance abuse (alcohol and marijuana) presented to the emergency department with complaints of bilateral lower extremity numbness and difficulty with ambulation, as well as a tingling sensation from the umbilicus to his toes. The patient denied muscle weakness on admission; however, the patient did admit to recreational nitrous oxide use and stated a recent increase in the amount of use. In addition, he started suffering from a similar episode around 10 years ago. In regard to this episode, he reported symptoms began 1 week prior to admission while inhaling nitrous oxide. Neurological exam was significant for the following abnormalities, with all other neurological tests being normal: (a) strength was 4/5 in both lower extremities proximally and distally and (b) loss of sensation from the umbilicus down to feet.

Clinical workup included MRI of the cervical and lumbar spine and lab work. MRI results showed mild degenerative disease in the thoracic spine. Lumbar spine changes were consistent with diffuse disc bulge at L5-S1 without significant foraminal stenosis. Moreover, cervical spine changes were also consistent with mild degenerative disease. MR of the brain showed no acute abnormalities, but changes were significant for mild cerebral atrophy. Dawsons' finger morphology was absent, with no periventricular lesions present. Labs on admission were significant for macrocytic anemia with a hemoglobin of (9.5) and an MCV of (112.8). Low levels of B12 were also noted. Additionally, MCH was 36.8, MCHC was 32.6, and RDW was 73.2. Of note, glucose levels on admission were 116. Urine drug screen was positive for cannabinoids. Hospital course was significant for improvement of neurological symptoms with cessation of NO use, treatment with B12 followed by instructions to continue PO B12 1000mcg daily for 12 days as well as folic acid supplementation at 1 mg every day for 14 days.

## 3. Case 2

**Figure 1:**
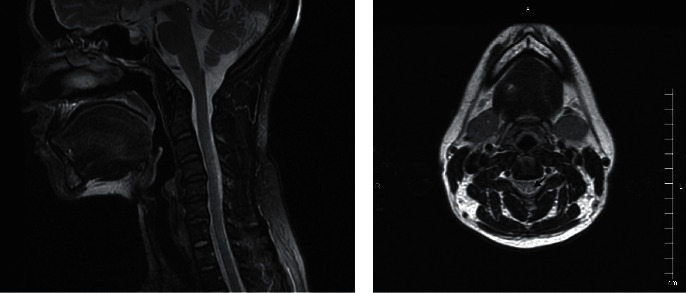
Case 2 MR C spine with and without contrast performed on 9/18/20 demonstrating a segmental abnormal signal within the dorsal columns of the cervical cord. (a) Sagittal cut T2-weighed imaging. (b) Axial cut T2-weighed imaging. Arrow: dorsal columns are prominently visualized and hyperintense.

A 36-year-old female with a past medical history significant for alcohol abuse, anemia, depression, pyelonephritis, and sepsis presented. The patient presented to the ED with tingling of both upper and lower extremities, lower extremity numbness, and a communicated history of recent trauma (fall from stairs). MRI during this initial visit revealed posterior column abnormalities with equivocal hyperintensities within the cord (Figure 1). The patient admitted to the use of nitric oxide for recreational use during the interview but denied any recent use (within 1 month). Labs on initial admission were significant for macrocytic anemia with a hemoglobin of 12.0 and an MCV of 104.1. Low levels of B12 were also noted, and initial MRI was within normal limits. Additionally, MCH was 34.9, MCHC was 33.5, and RDW was 82.3. Neurological exam was significant for decreased temperature and light touch sensation beginning from the lower rib cage to her toes. Reflexes were absent in both upper and lower extremities. Additionally, mild dysmetria was noted on the finger-to-nose and heel-to-shin test, bilaterally. The patient was diagnosed with subacute combined degeneration of the cord secondary to NO use, placed on IVIG for 5 days, and discharged with instructions to follow a regimen of PO B12 1000 mcg daily for 12 days.

Unfortunately, the patient's condition did not improve during hospitalization, and she presented to the ED a week later with primary complaints of knee pain associated with right lower quadrant abdominal pain that radiates to back and worse with movement as well as saddle anesthesia. The patient's numbness had progressed, and clinical workup showed continued hypovitaminosis B12, altered proprioception, and sensation. The patients' past medical history included drug-seeking behavior as well as alcohol abuse, another well-known cause of B12 deficiency and likely contributing factors to the unique and severe case presentation. For example, the patient also reported a continued sensation of rolling her ankles bilaterally, altered sensation from the neck down, and an insignificant improvement in gait. Hospital course was complicated by neurogenic bladder and bowel, orthostatic hypotension, dysphagia, insomnia, leukopenia, and neuropathic, as well as nociceptive pain. Clinical workup demonstrated an MCV of 106.2, hemoglobin of 12.3, homocysteine of 10.38, and folic acid level of 10.1. Following appropriate treatment with oral as well as monthly B12 IM injections in addition to folic acid supplementation for six months, the patient's neurogenic bladder was resolved. Additionally, after following a significant physical therapy regimen, the patient can now ambulate normally.

## 4. Discussion

The pathogenesis of vitamin B12 deficiency revolves around the liver's ability to provide for a significant vitamin B12 depot. Given the body's significant storage, vitamin B12 deficiency can be provoked acutely due to a severe insult or chronically through multifactorial events involved in vitamin B12 intake, absorption, or metabolic processing [3]. Depending on the cause for deficiency, symptoms may take as long as 10 years to present, as is the case with decreased intake, or dramatically within months, which may be seen with severe nitrous NO, as descried in the abovementioned cases [13]. Initial clinical presentation can be diverse but generally aligns with one of the following organ systems: hematologic, neuropsychiatric, cutaneous, or gastrointestinal [13]. Macrocytic, megaloblastic anemia and the associated symptoms of fatigue, pallor, or cardiac palpitations are considered the most common presentation, but a significant population of patients with vitamin B12 deficiency may present solely with neuropsychiatric symptoms, cutaneous changes, or disease of the tongue (glossitis) (Table 1) [13].

During the workup of hypovitaminosis B12, certain pathologies must be excluded. For example, the diverse array of symptoms related to hypovitaminosis B12 included nonspecific findings such as fatigue, depression, erectile dysfunction, paresthesia of the extremities, and muscle cramps, as well as cognitive disturbance [1]. Given the various neurological manifestations, neurologic conditions as well as other vitamin deficiencies must be ruled out, including vitamin D and folate, as well as hormonal deregulations such a thyroid hormone. Additionally, psychological causes of depression and fatigue, such as MDD and PTSD, must also be ruled out. A careful review of medications should also be a part of the diagnostic workup, given that commonly prescribed medications such as proton pumps inhibitors and metformin. as well as histamine 2 receptor antagonists, are found to be common culprits in the setting of B12 deficiency [16–18]. Cramps and paresthesia, the common complaints from patients with hypovitaminosis B12, are often due to electrolyte imbalances and must be explored and ruled out (Table 2). In recent years, there have been reports highlighting the unreliable uses of MMA and homocysteine as a marker of B12 status [19]. In response to a need for a more reliable screening marker, holotranscobalamin (holoTC) has received attention [19–21].

Regarding treatment of hypovitaminosis B12, those patients suffering from neurological symptoms must be differentiated from those which are not, as this changes the treatment strategy. For example, if neurological symptoms are present, it is recommended that intramuscular injections of cyanocobalamin be administered every other day for up to three weeks [13]. Conversely, if none are present, three times per week for two weeks of intramuscular injections of cyanocobalamin is the recommend course of treatment. Specific derangements will differentiate the recovery period, for example, neurological symptoms may take between six weeks to three months to improve, while anemias may take eight weeks [13]. Notably, if a concurrent folate deficiency is present, B12 deficiency must be repleted first in an effort to prevent subacute combined degeneration of the spinal cord. Lastly, in patients with an irreversible cause of hypovitaminosis B12, repletion should be continued indefinitely.

## Figures and Tables

**Table 1:** A comprehensive list of signs and symptoms associated with vitamin B12 deficiency organized by the organ system.

Constitutional Fatigue, depression	Cardiac Palpitations Chest pain	Reproductive Infertility	Pulmonary Shortness of breath
Cutaneous hyperpigmentation (maculopapular rash) Jaundice Vitiligo Pallor	Gastrointestinal glossitis (pain, swelling, tenderness, and loss of papillae)	Hematologic Anemia (macrocytic, megaloblastic) L eukopenia P ancytopenia (bone marrow suppression) thrombocytosis Elevated indirect bilirubin Elevated AST Decreased haptoglobin level Elevated MMA Elevated homocysteine Elevated LDH Thrombocytopenia	Neuropsychiatric Areflexia, Forgetfulness, Restless leg syndrome, Ataxia or positive R homberg test Extrapyramidal signs (eg, dystonia, dysarthria, and rigidity), Cognitive impairment (including dementia-like Cognitive slowing and acute psychosis), Gait abnormalities Depression or changes in mood, Irritability Lhermitte sign, Peripheral sensory deficits (loss of proprioception and vibratory sense) Olfactory impairment, Peripheral neuropathy Optic atrophy Anosmia Loss of taste

**Table 2:** Causes of vitamin B12 deficiency; representation of the exhaustive causes of hypovitaminosis B12 categorized by inactivation, malabsorption, and dietary deficiency.

Inactivation of B12	Malabsorption	Dietary deficiency
Use of nitrous oxide (most commonly in the recreational setting) Nonrecreational use of nitrous oxide anesthesia and underlying subclinical deficiency present	Pernicious anemia Gastrectomy, Gastric bypass, Ileal resection Inflammatory bowel disease, tropical sprue, Crohn disease, Tapeworm infection Transcobalamin II deficiency (inability to transport vitamin B12) Inherited disorders of impaired B12 metabolism Protein-bound vitamin B12 Malabsorption Medications (Metformin, Histamine H2 Blockers, and Proton Pump Inhibitors)	Diet (vegan or vegetarian) Alcohol abuse
